# Potato Ethics: What Rural Communities Can Teach Us about Healthcare

**DOI:** 10.1007/s11673-023-10242-x

**Published:** 2023-04-12

**Authors:** Malin Fors

**Affiliations:** 1 Finnmark Hospital Trust, Hammerfest, Norway; 2 UiT, The Arctic University of Norway, Hammerfest, Norway

**Keywords:** Rural, Geographical narcissism, Ethics, Disaster prevention, Pragmatism, Rural proofing, Potato ethics

## Abstract

In this paper I offer the term “potato ethics” to describe a particular professional rural health sensibility. I contrast this attitude with the sensibility behind urban professional ethics, which often focus on the narrow doctor–patient treatment relationship. The phrase appropriates a Swedish metaphor, the image of the potato as a humble side dish: plain, useful, versatile, and compatible with any main course. Potato ethics involves making oneself useful, being pragmatic, choosing to be like an invisible elf who prevents discontinuity rather than a more visible observer of formal rules and assigned tasks. It also includes actively taking part in everyday disaster-prevention and fully recognizing the rural context as a vulnerable space. This intersectional argument, which emphasizes the ongoing, holistic responsibility of those involved in rural communities, draws on work from the domains of care ethics, relational ethics, pragmatic psychology, feminist ethics of embodiment, social location theory, and reflections on geographical narcissism.

As a born urbanite, I have had to learn how to navigate a rural context during fifteen years of practice as a clinical psychologist in an Arctic community remote from urban centres. As I have come to understand both its unique demands and my own geographical narcissism (Fors 2018b), I have been struck by the insufficiencies of mainstream professional ethical standards. In this paper I argue for an ethics rooted in the duty to make oneself useful, an orientation I am calling “potato ethics.” The term appropriates a metaphor used in Sweden, where “*being a potato*” compares human activity to being a humble side dish: general, useful, and versatile—cooperating in the service of whatever is necessary and actively learning whatever is needed to do so. Potato ethics includes the moral obligation to make oneself useful, mentalize the context, and approach rural issues with a social justice perspective. Concretely, this means compensating for staff discontinuities that result from difficulties attracting long-term employees to rural areas, doing tasks that are distant from one’s training when there is no one else to do them, taking practical circumstances such as distance and weather into account, and working actively to prevent foreseeable disasters. Potato ethics refers to the intention to fully connect to the context while embracing and acknowledging that rural areas are colonized, vulnerable places that deserve both respect and what Tronto (2020) called *caring democracy* and *relational responsibility* (2012). Let me first review more classical views of medical ethics and suggest some limits of those perspectives.

## Classical Views of Medical Ethics

The field of medical ethics commonly reflects two major streams of influence: philosophical theorizing and clinical problem-solving (Simpson and McDonald 2017). To those I would add a social justice perspective, an ethical point of view that has emerged from clinical concerns arising in the context of the human rights movements.

### Medical Ethics Emerging From Clinical and Social Justice Perspectives

Most writing on medical ethics addresses either the doctor–patient treatment relationship or general public health issues such as access to healthcare. Concerns about access may be from a perspective of global health, such as fair vaccine distribution, or may involve attention to unfair prioritizing in specific clinics or countries. The primary focus of professional guidelines—for example, the Code of Ethics of the American Psychological Association (2017), the Ethical Guidelines for Psychologists in the Nordic Countries (Sveriges Psykologförbund 1998), and the Ethical Guidelines for Physicians in Norway (Legeforeningen 2021)^1^— has been on the doctor–patient or scientist–subject relationship, including such areas as avoiding harm, refraining from exploiting the patient, and conducting ethical research. Common topics include concerns about what kinds of decisions and choices patients can understand and make (e.g., when is consent considered truly “informed”) (Agledahl, Førde, and Wifstad 2011), patient access to medical records (Fors and McWilliams 2016; Walker, et al. 2014, 2011), and social justice perspectives on care that are sensitive to power differences, racism, and ethnic and cultural factors (e.g., Drescher and Fors 2018; Fors 2018a, 2018b, 2019, 2021a, 2021b; Kirmayer 2012). Other emphases concern access to care and discrimination in allocating resources.

### Medical Ethics Emerging From Philosophy

Medical ethics questions that have arisen from philosophical inquiry involve inferring moral principles that inform imagined clinical situations. For example, two patients need a heart transplant but only one heart is available. The person who gets it will survive; the other will not. Who should have the heart? From a utilitarian perspective, the Swedish philosopher Tännsjö (2021a) suggests that the person with the greatest amount of probable future happiness should have the heart. That solution would give the organ to a younger person rather than an older one if both have the same underlying health issues and prognoses because the young person has more predicted years of living. An egalitarian logic would argue instead for equal chances of getting the heart, perhaps suggesting the drawing of lots to solve the problem (cf. Tännsjö 2021a). In a response to naive egalitarism, Tännsjö (2021b) wrote: “I don’t want to pull my kids out of the line for intensive care.”^2^ He argues that some forms of egalitarianism represent a denial of extreme situations in which prioritizing is necessary; implicitly equating old persons and children by drawing lots would be unreasonable. Recently in the COVID pandemic, Tännsjö (2020) argues that other possible ways to prioritize include helping the many in preference to the few (for example, saving five eighty-six-year-olds old rather than one twenty-two-year-old), and giving priority to healthcare personnel^3^—not because they are entitled or superior in any intrinsic way but because their work puts their lives at risk and because it is of value to the community to keep doctors and other medical workers alive.

From a philosophical perspective, other medical ethics questions have been considered. What about terminal sedation? Is it euthanasia in disguise (Tännsjö 2004)?

Why is euthanasia seen as morally acceptable in some countries but not others (Healey 2017)? And if one accepts euthanasia, what problems arise when offering this possibility to psychiatric patients? (see, e.g., Nicolini, et al. 2020). Other pressing ethical questions have been raised in the areas of xenotransplantation, DNA selection for in-vitro fertilization, selective abortion, and cloning.

### Limits of Traditional Medical Ethics

All the themes above are of course important and constitute crucial issues in medical ethics. However, none of these angles of vision expresses the social justice perspective that frames the doctor or nurse or psychologist as a caring agent in society, with a task that goes beyond the doctor–patient relationship, beyond philosophical theorizing, and beyond issues of access to care or patients’ rights to be treated without discrimination. A potato ethics perspective assumes that health personnel have responsibilities that are broader than issues like informed consent and other doctor–patient matters. They have a duty to serve society as a whole.

This larger ethical sensibility has been evident in public policies such as Norway’s decision on March 12, 2020, to close the nation during the pandemic and *forbid all health personnel to leave the country*. Although such fiats are rare, in this case Norway relied on what I am calling potato ethics in insisting that their doctors, nurses, psychologists, laboratory personnel, and associated medical professionals go beyond their own individual interests and job-definitions to serve the community. The citizens were in a vulnerable situation, and everyone who could help was obligated to do so. Interestingly, Norwegians responded to this decision approvingly and with pride. There were no debates about restricting individual freedom, no arguments comparing Norway to a communist dictatorship; instead, a broad unanimity prevailed. Doctors who learned of the press release while waiting to board planes at Oslo’s Gardermoen Airport cancelled their flights and returned home without complaint.

## Potato Ethics — An Imperative to Be Accountable and Useful

What I am calling potato ethics involves the moral imperative to make oneself useful, to mentalize one’s context and see that context as an important part of a larger social justice picture. Here I suggest a switch of perspectives away from narrower definitions of professional ethics. Instead of looking only at what rights patients should have or considering what errors a doctor might make in a clinical situation, I am addressing here ethical duties that are at the core of being a professional, duties that are inherent in choices about where and how to serve and contribute (or not). As an agent with the power of knowledge, the doctor has an obligation to use that power ethically and for the maximum benefit of the community. Rural realities offer a good example of why potato ethics matter, but there are no limits to the situations in which the logic of potato ethics can be applied. A potato ethics perspective upends certain basic assumptions of healthcare ethics and makes the doctor an *accountable participant in the community*. It underscores the responsibility that comes with the trust that vulnerable people put in health professionals; it goes beyond the discussion of competence, technical priorities, and narrow doctor–patient interactions. It is an ethic that takes into account power, space, and time (Fors 2018b; Winther 2014) and embraces the philosophy of pragmatism (Fishman 1999; Goldberg 2002) as well as what has been called the ethics of ordinary life (Das 2012). Being able to be radically pragmatic results in saving lives in rural areas. In construing the physician as an accountable agent in vulnerable settings, potato ethics formulates an alternative ethical language that goes beyond relegating the doctor to the role of private person whose career plans reflect only personal, individual interests. A potato ethics perspective emphasizes relationality and dependence and underscores the responsibility that comes with the trust that lay people invest in health professionals.

### Relational Ethics

A core value of potato ethics is its *relational* orientation (Tronto 2012). It calls for a personal moral duty beyond professional rules and professional ethics. It operates as a parallel to the fact that in effective psychotherapy, in addition to the professional relationship and the transference, there is an authentic alliance between two human beings. This assumption is central, for example, to Gabbard’s (2017, 59) ethical reminder to young therapists: “When in doubt, be human.”

Gentile (2013, 2017) argues from a relational psychoanalytic and feminist standpoint that witnessing is never enough; we are always a part of the context ourselves, and in that sense, social justice issues pervade psychotherapy encounters. Harding (2004, 2009) has memorably argued that our social locations even form how we experience the world itself; she attributes a “stronger objectivity” to people in subordinated groups. In psychotherapy and medical relationships, privilege is hardly undone or wiped out. In an earlier text (Fors 2021a) I have argued that some privileges are not optional: a white person embodies white privilege irrespective of how “innocent” or “goodhearted” (in itself a naive idea) that person is. White privilege would operate as a silent benefit in any situation because of its meaning in the larger social context. Sara Ahmed goes so far as to call whiteness a “bad habit” (2007). The benefits of inherited privilege come with what Esquith (2010) has called a bystander responsibility. Suchet (2007) addresses this issue in poignant and painful ways via her account of growing up in South Africa with a black nanny; her story exemplifies how skin colour is never innocent, painless, or colourblind.

Tronto, a professor in political science and women’s studies, (2012, 311) writes:

Ignorance has many meanings, but consider its role in asymmetric relationships. When the more powerful get advantages by ignoring the effect of the relationship on the less powerful, their ignorance proves beneficial.

Prilleltensky and Nelson (2002) have made comparable arguments for the political duty to use psychology for social justice purposes. Rural realities include continual reminders of human vulnerability. They showcase how damaging ignorance can be. If one is, for example, the only available doctor, one’s responsibility is both profound and impossible to ignore. Living in rural communities unquestionably changes one’s standpoint (Harding 2004, 2009) and makes one aware of one’s own geographical narcissism (Fors 2018b).^4^ Tronto (2012) underscores the harm of irresponsibility:

Once we begin to notice that assigning, accepting, deferring, deflecting, and meeting responsibility involves power, some of the important asymmetries of responsibility are revealed. The advantage of the relational approach to responsibility becomes clear once we begin to think about assessing the seriousness of irresponsibility. … Some elements become more important in assessing the harm of irresponsibility. (308)

I am suggesting a relational dimension here that goes beyond formal or classically construed professional ethics. Reader (2003) suggests that *presence* calls for a certain form of responsibility: “…[P]resence constitutes obligation: we recognise that when someone collapses in front of me, I am obligated to help them by the real connection between us that is our presence to each other” (Reader 2003, 372).

The potato ethics I am describing could be applied to other spaces and contexts. Consistent with Tronto’s (2020) argument that care ethics (Gilligan 1982) must be widened to address sociopolitical dimensions, I posit that rural–urban tensions are inherently sociopolitical. *Presence*, in a rural area, involves sharing the same space and encountering suffering without the distance of limiting oneself to making transference interpretations or voicing complaints about remote bureaucracies or enjoying the advantage of limiting one’s competence to a narrow area so as to avoid extending oneself into uncharted clinical territory. I suggest that the urban–rural tension needs to be a part of the power questions we raise in intersectional analysis (Crenshaw 1989; see also Fors 2018a, 2018b) and that that tension (or colonization, Fors 2018b) accentuates the question of actively making oneself useful. When we are sharing the same space (Fors 2018b; Winther 2014), sharing presence, being more obviously connected to one another (Reader 2003), and seeing the raw consequences of ignorance (Tronto 2012), then witnessing without feeling (Gentile 2013, 2017) becomes impossible. Tronto asks:

How do we change our concepts about humans so that instead of thinking of them as autonomous, we also recognize them as vulnerable and interdependent? … To do so, we have to re-imagine democratic life as ongoing practices and institutions in which all citizens are engaged. This engagement presumes that relational selves, who need ongoing participation as both receivers and givers of care, will be central in making judgments about responsibility. (2020, 169)

## Rural Realities

In rural settings, clinicians often find themselves to be the only option for the patient. In connecting an intersectional analysis with an ethic of care, I am indebted to the work of Hankivsky (2014), who stresses:

A distinguishing feature of care ethics is the recognition that humans are concrete beings, who exist in mutually interconnected, interdependent, and often unequal relations with each other. What makes care ethics so compelling is its view that “all people are vulnerable, dependent and finite, and that we all have to find ways of dealing with this in our daily existence and in the values which guide our individual and collective behaviour” (Sevenhuijsen 1998, 28). Care places at the forefront human flourishing and the prevention of harm and suffering. (253)

This is a kind of care ethics but expanded beyond its original conceptualization.

...[C]are ethics may benefit from the theoretical insights of intersectionality, but it is important not to lose sight of the fact that the reverse can also be true. For all its attention to expanding on understandings of the intersecting factors that shape and determine inequity, it is also the case that most intersectionality scholars have not paid much attention to care as a practice that shapes human lives. More explicitly, they have not grappled with how dependency and vulnerability are linked to care, nor have they confronted the fact that everyone, regardless of their social location, at one time or another will receive or give care. (Hankivsky 2014, 262)

So, what do intersectional care ethics mean for the rural? How could we include attention to the rural as a dimension in power analysis? Although many texts on rural health issues emphasize rural deficits (for a critical overview, see, e.g., Bourke et al. 2010; Simpson and McDonald 2017; Walkerman 2008), and thus focus on specific challenges to healthcare such as long distances, bad weather, rigid religious attitudes, low intelligence, lack of education, and challenges in recruitment and retraining (Aten et al. 2010; Boilen 2021; Curtin and Hargrove 2010; Smalley et al. 2012), a different and more critical perspective has recently emerged.

### Social Justice in Rural Medicine

Simpson and McDonalds (2017) state that there has been insufficient attention to rural health ethics and that urban bias includes medical ethics formulations. In an earlier paper I suggested the term “geographical narcissism” (Fors 2018b, 446) for the urban tendency to be self-centred, to hold “the common but unconscious beliefs that space, time, and power emanate from the urban world.” Gundersen (2021) called the unfair distribution of health services favouring urban areas “geography-based discrimination.” Others have called it “structural urbanism” (Probst et al. 2019) or urban bias.

Couch et al. (2020), after interviewing twenty-five general practitioners based in rural Tasmania, found that rural clinicians were invested in the next generation. They thought about future developments, future power issues, and the future of the rural space they were serving (Fors 2018b; Winther 2014). Other positive formulations come from the paradigm of clinical courage (Maclellan 2011; Wootton 2011; see also Konkin et. al. 2020).

Accordingly, there have been some notable pioneers dedicated to social justice and harm reduction in rural medicine (e.g., Husum et al. 2003; Wisborg et al. 2003, 2008). For example, Wisborg and colleagues educated lay people to do first aid, thereby increasing the survival rates for people in rural Cambodia and northern Iraq who had suffered accidents from mines. They created a “Village University” to educate 135 local “paramedics” and 5200 “first responders” to give advanced first aid in rural places far from the hospital. Death rates decreased from 40 per cent to 14.9 per cent, a change that affected the society as a whole (Wisborg et al. 2008). In rural Ghana, Mock et al. (2002) demonstrated the high impact on public health of giving taxi drivers a six-hour training course in first aid. These projects express the ethics of pragmatism. The scientists who were involved honour the autonomy of those in rural areas rather than behaving as experts or missionaries who may make people dependent and then leave the stage. Those examples serve as a reminder of treating rural vulnerable spots with respect and dignity and with an anti-colonizing agenda.

## Potato Ethics

Truly seeing the context, and being aware that sometimes one’s own limited expertise is the only option for a patient, expands professional responsibility. In Benjamin’s (2017) terms, to *recognize* the other as well as oneself, is to be radically aware of human vulnerability.

### Being Accountable to Contribute

I live in Hammerfest, Norway, the northernmost town in the world. I am the only psychologist with a private practice within a two-and-a-half-hour driving distance. My specialties are long-term psychotherapy, psychoanalysis, and psychodynamic psychotherapy.

One day the manager of the local dental clinic called, saying they needed a psychologist to do assessments for odontophobia. I declined, saying “Well, you will have to ask someone else. I don’t do CBT.” 5 They called to beg: “Please. We have no one else to ask.” I turned them down again, politely explaining that the treatment of odontophobia was not within my scope of competence. When they called me a third time, I finally felt their vulnerability. They were not exaggerating; I was their only option, and it would be selfish not to try to help. It turned out that what I thought would be boring exposure therapies were actually very meaningful trauma treatments. Helping patients who had been in dental pain for many, many years was immensely satisfying. I felt some shame for not having agreed to help the dentists the first time around. Although it was not my specialty, it did not cost me many calories to learn the relevant techniques well enough to be of help (see also Fors 2021b, 2022). In retrospect, I think my own geographical narcissism made me blind to the real needs of my community and to my accountability as a trained psychologist when a community asks for help.

My point is that in a rural setting, one must be constantly aware of one’s deficits and areas of incompetence and yet still willing to learn how to be of use. In an effort to respond to this reality, the American Psychological Association has suggested that in the absence of other resources, one *may* provide services that are beyond one’s usual competencies:

Psychologists provide services, teach, and conduct research with populations and in areas only within the boundaries of their competence, based on their education, training, supervised experience, consultation, study, or professional experience … When psychologists are asked to provide services to individuals for whom appropriate mental health services are not available and for which psychologists have not obtained the competence necessary, psychologists with closely related prior training or experience may provide such services in order to ensure that services are not denied if they make a reasonable effort to obtain the competence required by using relevant research, training, consultation, or study. … In emergencies, when psychologists provide services to individuals for whom other mental health services are not available and for which psychologists have not obtained the necessary training, psychologists may provide such services in order to ensure that services are not denied. The services are discontinued as soon as the emergency has ended or appropriate services are available. (APA Ethical Principles of Psychologists and Code of Conduct, 2.01.)

Note that this ethical principle is not formulated as *a duty* to contribute, to learn, or to try to achieve the competence needed for the society one lives in. Rural situations are implicitly formulated as *exceptions* (Fors 2018a, 2018b) to an urban ethical norm, itself a form of urban arrogance or geographical narcissism. Such formulations may be seen as a subtle version of dominant groups’ tendencies to project unacceptable behaviour onto othered groups, such as framing black people as lazy, Jewish people as greedy, gay people as promiscuous, or women’s bodies as weak (Fors 2018b). There is no stated ethical duty in the APA ethics code for urban professionals to help rural professionals design courses, educational programmes, or on-line supervisory opportunities (e.g., Østmo, Jørund and Fors, paper under peer review). I am aware of only this rare exception: In Norway, to get fully licenced as physicians, all students graduating from medical school are obligated to do a six-month internship as a general practitioner wherever the Norwegian Directorate of Health assigns them. The final destination, or “rural duty” (distriktsturnus), is decided by drawing lots (Ministry of Health and Welfare 2001).

As I reflected on my having turned the dentists down twice, I began to unpack the political magnitude of the situation. The historical lack of qualified dentists had contributed to the suffering of odontophobic patients. As I noted in another paper (Fors 2022):

A special form of rural odontophobia developed here [in Hammerfest] because of a combination of a lack of trained people and lack of control. Dentists who couldn’t get a job anywhere else would come here. Some were drunk while they were working; some worked without providing pain relief. (40)

Being in a rural area is to be constantly in touch with deficits, trauma, and the far-reaching consequences of colonization. Accordingly, Shank (1998) shares the voice of one health professional he interviewed:

I have practiced outside the scope of my license a million times since I have been here because I sometimes feel like something is better than nothing … I was also working in a system where I have to see them, or no one else would take them. It wasn't even a matter of referring. (275).

### Building Continuity as the Elf

Compensating for the lack of resources or competence is another part of potato ethics. The tendency of many psychologists, doctors, and nurses to narrowly guard what is their own task, and not to become anyone else’s assistant, is not consistent with potato ethics. Potato ethics requires one to make oneself useful, even if the task is not glamorous. This may mean babysitting doctors who rotate in and out of one’s service, assisting them with administrative demands, pre-reading medical records to summarize them for visiting specialists, pointing out crucial assessment dates and documents, enabling medical professionals to order lab tests from a general practitioner, or writing letters that can be signed only by them.

Many urban physicians come to Hammerfest on very short contracts, sometimes only for a few days. They sometimes create more problems than they solve. A colleague summed up his wisdom about this situation in a series of metaphors: “We are like the invisible elves sewing all the holes, preventing errors of discontinuity. We are catching all the balls that are going in different directions. We are invisible, but we provide the safety net for patients.”^6^ Local professionals are often experts at anticipating pitfalls and potential dangers of systems on which rural communities have to rely (eg. Harbitz et al. 2021).

Extraordinary problem-solving is an everyday task of rural nurses faced with a lack of recourses. From the perspective of being a patient myself, I want to share this story:

I had a mysterious eczema covering my whole body and started three-times-a-week light treatment at the local hospital. After the first week, my skin was swollen, and I was afraid to continue the treatment without checking with a dermatologist. The nurse was worried too, but she said the only dermatologist in the whole county was off work for a month. I had to choose whether or not to quit treatment while I waited. She called me soon afterward, saying she had fixed the problem. She knew a teaching dermatologist who would be coming to lecture the medical students in a few days. He was not hired by the hospital but by the university, but he had offered to see me pro bono, since the procedure for paying his clinic in the south of Norway would be a bureaucratic nightmare. He lowered the dose of the light treatment, corrected the diagnosis, and called me three times to follow up. The eczema disappeared. Going outside the rules saved me from weeks of terrible itching. When I tried to thank the local nurse for her thoughtfulness and creativity in helping me, she minimized her role: “Of course we try to help, always.” To her, thinking “outside the box” was normal. Her professional pride and personal self-esteem depended on potato ethics.

Sometimes potato ethics means breaking rules for reasons that take into account the context and the particular vulnerability of the patient. I remain grateful that my nurse was able to intervene with my problem and that the visiting dermatologist teacher was sufficiently attuned to the context to help me. He helped me only because he could and because he realized I was in need.

### Being Pragmatic

Where rural ethics are not emphasized, people use urban standards to solve rural problems, often with a naive idea that solutions provided by urban bureaucracies will solve similar problems in rural settings. Urbanites tend to have a positive Weberian (Weber 1997/1920) attitude toward record-keeping, forms, referrals, and bureaucratic procedures, while people in rural communities, especially when crucial positions are not staffed, often encounter Kafkaesque versions of bureaucracy. Clegg and colleagues (2016) note:

While Weber suggests the inevitability of the technical superiority of forms and describes the attendant “iron cage” that it produces, Kafka spoke from within this cage, telling dark and enigmatic stories of the ironic futility of bureaucratic life. While Weber told us about bureaucracy’s rationality, Kafka led us through its dark labyrinth. While Weber wrote about the impersonality of bureaucracy, Kafka vividly evoked the lived experience of its supplicants being constantly confounded by its machinations. (157)

Using creative interventions to address rural challenges is consistent with the philosophy of pragmatism (Fishman, 1999; Goldberg 2002).

Pragmatism is seen as a theory of instrumentation or a collection of tools for accomplishing goals; it claims that many of our efforts to know and seek truth are based upon myths … Pragmatism advises us to focus on the possibility that we may be captured by one or another of the above mentioned myths as we struggle to resolve the unresolvable. Knowledge must be seen as a tool for adaptation, rather than as a picture of reality. (Goldberg 2002, 235–248).

### Potato Ethics in Negotiation with Naive Urban Assumptions

Being pragmatic in situations where others would regard pragmatism as a lazy shortcut may stir up problematic feelings among busy staff members.

Yet another urban satellite psychiatrist was visiting our rural clinic. As had happened many times previously, members of our team felt he was “urbansplaining” to us.^7^ We had come to find this recurrent experience oddly entertaining. The psychiatrist was shaken. *No one had done things correctly*. Rural places seemed to see themselves as entitled to invent their own rules, he warned. This was NOT ethical. I tried to respond to his critique by formulating rural ethics in terms of pragmatic adaptations to situations that are radically different from those assumed by urban bureaucracies. I suggested that procedures that had been established to address urban concerns should not override the reality principle (Freud 1900). A nurse on the team sent me a secret smile. I stiffened up and tried not to meet her eyes for fear I would start laughing.

The theme of the heated conflict was whether or not to write a formal “specialist statement” to a patient’s general practitioner to use for her application for a disability pension. Everyone agreed that this patient needed a disability status; the difference of opinion involved formalities. The doctor in question had not asked the clinic in the correct manner for a specialist statement (a letter that had to be from a psychiatrist or a psychologist) because the local NAV office (the Norwegian Labor and Welfare Administration) *had not sent the correct request* yet on the correct form. This meant that our clinic would not be able to charge NAV for the specialist statement. Because our hospital is public, and NAV is a public insurance, it was the same governmental money, held in different pockets; not getting paid did not hurt us personally. What the psychiatrist suggested was indeed the correct procedure: according to the rules, NAV should send us a form and pay a fee for each statement. The problem was that despite many requests, no NAV officials had done so for at least fifteen years — my tenure at the clinic. The local NAV office had a high employee turnover rate, a relatively uneducated staff, and a shifting leadership. They tried to keep current with laws on disability money, social welfare, sickness leave, and other employment issues; they even organized air travel to orthopaedists for people needing arch support, to a town thirty-five minutes away by plane. So naturally they were incompetent in some areas and not updated on this relatively obscure regulation. Their stated position was that they would request a specialist statement *only when they needed one to conclude a case*. In reality, this meant never. The only way to intervene professionally to influence their decision on our patients’ behalf was to send *uninvited specialist statements*. Everyone in our rural area accepted this local way of navigating NAV. This was what the general practitioner was asking for now, for us to help the patient by sending a specialist statement even though no form was in our hands. NAV had the power to decide disability status on the basis of limited information but they did not have the power to neglect a specialist statement sent from us, even if unrequested. So here we were.

The urban psychiatrist was technically right. The problem was that he assumed that we could inform NAV of the relevant law, and that our educational efforts to that effect would have an impact. He implied that we were behaving unethically. I had become resigned long ago to the realities of our situation. I had long ago decided that if my boss did not fight NAV every time they failed to send a bill, I would not bang my head against her unwillingness to confront them. I had seen many patients hurt by NAV’s failures: deserving people who did not get money that they were due, making them unable to pay their bills. I thought being pragmatic was more ethical then being correct. The psychiatrist was shaken. I was angry. But I also felt ashamed: I could have made this case fifteen years ago. Living in rural vulnerability changes the way one perceives the world.

The ethics were complicated. Did we contribute to resignation, anarchy, and disrespect for important bureaucratic rules? Did our behaviour further undermine NAV’s competency? Or were the bureaucratic rules imperfect for rural realities (Clegg, et al. 2016)? Did we navigate a whole different reality, not only embracing the reality principle (Freud 1900; Sheehi 2020) but, more important, preventing disasters. I called a friend in the field of medical ethics to sort this out. Was my position unethical? Did ethical standards matter more to the psychiatrist than to me? Or was the reading of consequences our main disagreement? If the psychiatrist had seen what I had seen, would he change his mind? If he were able to visualize patients without money, getting sicker, enduring one after another fruitless meeting with NAV, being disappointed every time they were refused, having to mobilize enough energy to take up the fight again, putting up with yet another short-term psychiatrist taking over their case — would he still find me unethical? We were trying to avert a foreseeable disaster, while he was following an urban rule.

### The Ethics of Disaster Prevention

Cook and Hoas (2008) note that the vulnerability of organizations that depend on travelling health personnel increases when visiting professionals are insensitive to rural realities. In a previous article, I mentioned the rural problem of dealing with urban “leftovers” (Fors 2018b); that is, urban professionals who come to work in rural areas when they have been unable to find a job anywhere else. In such instances, local professionals sometimes have to compensate or minimize damage. Cook and Hoas (2008) describe the work of a pharmacist in a rural area who would correct a physician’s prescriptions. Vernillo (2008) sums up the ethical problem described by Cook and Hoas (2008):

(When) The pharmacist . . . . corrects a prescription that has the wrong dosage, an ethics quality gap exists whereby systems or processes designed to promote ethical practices are not functioning well. A pharmacist cannot legally change a physician’s prescription without permission from the physician. However, the physician tends to “slam the phone in your ear” when his orders are questioned or need clarification (Vernillo 2008, 62).

In other words, potato ethics includes disaster prevention. This activity may provoke ethical collisions with visiting urban colleagues, who may not fully comprehend the implications of certain rural deficits. Local health professionals often know they have to intervene to avert disasters (e.g., Sakakushev 2021), and yet they may be realistically dependent on an urban psychiatrist who is committed to following national professional rules. Chambliss (1996) notes:

The great ethical danger, I think, is not that when faced with an important decision one makes the wrong choice, but rather that one never realizes that one is facing a decision at all. (59)

In a discussion of the Challenger space shuttle disaster, in which six astronauts and one teacher were killed, Harris (1995) emphases three sources of failure: *bad management, bad engineering*, and *bad ethics.* As it turned out, the engineers knew about a technical problem but were afraid to let management know. In discussing what he calls *preventive ethics*, Harris (1995) discusses the complications of avoiding disasters before they happen. Even though one person’s lack of disability money is not comparable to the deaths of seven people and the loss of a spaceship, the principle is the same. In navigating the ethics of preventing a micro-disaster such as a personal financial problem, the disaster would not occur in the absence of both a technical problem by the administrative officer at NAV and bad management at hospital, as evidenced by its having successfully addressed the problem for at least fifteen years. Relying on the only option left, our rural health team had invented a system to execute disaster-prevention ethics. From experience, we knew the likelihood of patient suffering if we were to persist in asking NAV for the correct form before helping the patient. Sakakushev (2021) formulates:

The challenge of disaster preparedness is how we give the best care possible under the worst possible circumstances. Lessons learned from disastrous past incidents could improve preparedness relying on better understanding and expertise. (577)

Much of the work of rural health professionals involves being constantly in the “worst possible circumstances.” In fact, rural health personnel often live under the ceaseless stress of anticipating disasters. To fully appreciate the vulnerability of rural areas is also to fully acknowledge unfairness in access to healthcare (e.g., Riksrevisjonen 2021; see also Sheehi 2020). As Farmer (2012) sardonically noted, “Rural is not simply urban with trees and animals.” According to Karadag and Hakan (2012, 603), the predisaster phase is crucial in ethical assessment: “Today, developing strategies to prevent disasters or to decrease the magnitude of disaster related injuries and damages is regarded as an ethical responsibility.” (See also Pikoulis, Pikouli, and Pavlidou 2021).

Cook and Hoas (2000) write about the implications of insufficient personnel for both clinic staffing and participation on ethical boards in rural healthcare settings. They investigate the loneliness and organizational problems that add to the rural burden “when the rubber meets the road.” Boilen (2021) suggests that being a rural clinician comes with a special risk of feeling overwhelmed, since we know rural communities suffer from greater needs and fewer providers.

In light of their observations, and those of others who have commented on burnout in rural settings, I want to state the obvious: potato ethics means serving a community, but it requires surviving oneself and finding one’s own ways to be nourished and to continue to grow as a professional. This is why for urban people, potato ethics implies, among other things, being accessible to rural colleagues. I conclude this essay with a plea to urban professionals in my field: when your rural colleague phones for advice, take the call. When your rural colleague wants to attend a course or meeting online, open it up for digital attendance. And when you have a dilemma to solve yourself that you cannot find the answer to in a textbook, call your rural colleague. Rural clinicians are among the most experienced people at solving extraordinary problems that no textbook has ever covered.

## Summary

What I have called “potato ethics” is an ethic of care (Gilligan 1982) as well as an ethic of democratic care (Tronto 2012, 2020). It builds on a relational model of responsibility (Tronto 2012) that prioritizes being useful to interdependent, vulnerable human beings. It connects Hakivsky’s contributions on intersectionality with care ethics (2014) but goes beyond these in referring to a professional health ethic that is not narrowly about doctor–patient or scientist–subject relationships. Potato ethics consider the whole social context, applying both an ethic of caring for democracy and a radical community-based ethics focused on outcomes. I have tried to demonstrate the superiority of this ethical sensibility to an ethic of rules that have been made with an urban reality in mind. It focuses on pragmatism instead of bureaucracy; it mentalizes power, space, and time. It recognizes vulnerability and honours probable consequences ahead of the rules that were designed to attain similar consequences in a different setting. It orients us toward avoiding micro-disasters and includes our willingness to be invisible, non-glamorous “elves” doing damage control. While potato ethics may provide a kind of natural heartbeat for health personnel in rural areas, its essence is about social justice and may be applied to any context. I am suggesting here that urban professionals have a lot to learn from rural colleagues, not only so that they can help them out but also because it may benefit them to reframe their own work in ways that go beyond their individual career paths. Potato ethics would ask that they embrace a duty and commitment to serve current and upcoming generations of patients and colleagues and come to an ethical stance organized around the imperative to be useful. When considered in the context of particular meals, potatoes are almost endlessly adaptable. By thinking about the larger context of their work, each professional might develop an appropriate personal recipe for ethical practice. Honour the potato.

## Footnotes

1 My own context in this matter is being a clinical psychologist in Hammerfest, Norway, working for a hospital, in private practice and teaching upcoming physicians for the medical school UiT, The Arctic University of Norway.

2 My translation.

3 In general, many people would feel intuitively that younger patients should have priority over elderly ones. They might, however, not feel intuitively comfortable turning off a ventilator for a fifty-year-old patient in recovery when a younger patient comes to a medical facility needing that equipment. In that situation, the patient who was there first tends to be given priority. This is common question addressed by medical ethicists.

4 An old mentor of mine said: “My regret after a long career in a rural area is not having dedicated myself to trauma treatment. If I were young today, I would do that.”

5 CBT is Cognitive Behavioural Therapy, commonly used to treat odontophobia with exposure therapies.

6 Thanks to Sven-Are Halvorsen.

7 “Urbansplaining,” which alludes to the term “mansplaining,” was coined by the rural Canadian physician Ken Milne. (See also https://thesgem.com/2019/04/sgem253-everybodys-working-on-the-weekend/.)
